# Prevention of Common Mental Disorders in Employees. Perspectives on Collaboration from Three Health Care Professions

**DOI:** 10.3390/ijerph15020278

**Published:** 2018-02-06

**Authors:** Eva Rothermund, Martina Michaelis, Marc N. Jarczok, Elisabeth M. Balint, Rahna Lange, Stephan Zipfel, Harald Gündel, Monika A. Rieger, Florian Junne

**Affiliations:** 1 Department of Psychosomatic Medicine and Psychotherapy, University Hospital Ulm, 89081 Ulm, Germany; marc.jarczok@uniklinik-ulm.de (M.N.J.); elisabeth.balint@uniklinik-ulm.de (E.M.B.); harald.guendel@uniklinik-ulm.de (H.G.); 2 Leadership Personality Centre Ulm, Ulm University, 89073 Ulm, Germany; 3 Institute of Occupational and Social Medicine and Health Services Research, University Hospital Tübingen, 72074 Tübingen, Germany; martina.michaelis@med.uni-tuebingen.de or michaelis@ffas.de (M.M.); rahna_shahriari@gmx.de (R.L.); monika.rieger@med.uni-tuebingen.de (M.A.R.); 4 Research Centre for Occupational and Social Medicine (FFAS), 79098 Freiburg, Germany; 5 Department of Internal Medicine VI, Psychosomatic Medicine and Psychotherapy, University Hospital Tübingen, 72076 Tübingen, Germany; stephan.zipfel@med.uni-tuebingen.de (S.Z.); florian.junne@med.uni-tuebingen.de (F.J.)

**Keywords:** occupational health physician, primary care physician, psychotherapist, depression, anxiety, health services research, outpatient care

## Abstract

Collaboration among occupational health physicians, primary care physicians and psychotherapists in the prevention and treatment of common mental disorders in employees has been scarcely researched. To identify potential for improvement, these professions were surveyed in Baden-Württemberg (Germany). Four hundred and fifty occupational health physicians, 1000 primary care physicians and 700 resident medical and psychological psychotherapists received a standardized questionnaire about their experiences, attitudes and wishes regarding activities for primary, secondary and tertiary prevention of common mental disorders in employees. The response rate of the questionnaire was 30% (*n* = 133) among occupational health physicians, 14% (*n* = 136) among primary care physicians and 27% (*n* = 186) among psychotherapists. Forty percent of primary care physicians and 33% of psychotherapists had never had contact with an occupational health physician. Psychotherapists indicated more frequent contact with primary care physicians than vice versa (73% and 49%, respectively). Better cooperation and profession-specific training on mental disorders and better knowledge about work-related stress were endorsed. For potentially involved stakeholders, the importance of interdisciplinary collaboration for better prevention and care of employees with common mental disorders is very high. Nevertheless, there is only little collaboration in practice. To establish quality-assured cooperation structures in practice, participants need applicable frameworks on an organizational and legal level.

## 1. Introduction

Common mental disorders like depression, anxiety, somatoform and adjustment disorders are highly prevalent globally [1]. Depression is the second most frequent cause of years lived with disability worldwide, as reported by a systematic analysis in 188 countries between 1990 and 2013 [2,3]. Approximately 15–20% of the working population suffer from a mental disorder at any one time, as reported by a survey in Western European countries, North America and Australia in 2012 [4].

The World Health Organization World Mental Health Survey collected data on the *inter alia* 12-month prevalence of major depressive disorder and the proportion of the latter receiving treatment between 2001 and 2015 [5]. In 2017, the results of 21 high, upper-middle and lower-middle income countries were published [5]. They show that, similar to other high income countries, only 20% of those afflicted with a mental disorder in Germany utilize the health care system [5,6]. Up to 50% of untreated common mental disorders can remit spontaneously within a year [7,8]. This questions the call for early interventions, as it might be a good strategy for some individuals [7]. However, affected individuals often experience social damage, like worsening of the social climate among colleagues or with supervisors due to reduced work performance [9]. 

Data collected between 2009 and 2012 within a comprehensive study in Germany revealed a median delay between the appearance of the illness and evidence-based treatment of six to seven years among individuals who did not make treatment contact in the first year [6]. Possible reasons are fear of stigmatization [10], long waiting periods for outpatient psychotherapy [11], regional differences in availability [12,13] or problems with referrals for psychotherapy treatment. Possible difficulties are the non-detection of the mental disorder or a lack of knowledge of additional health care options [14,15]. 

Collaboration across disciplines, services and sectors [16] has been valued as a crucial factor in improving mental health care from a practical [16,17,18,19] and political [20] perspective.

Efforts to improve collaborative care for individuals with common mental disorders in the workplace are promising. One study by Vlasveld et al. [21] has not shown a better continuous outcome of the depression score, but a greater response rate to the intervention compared to the usual care offered. A research group in The Netherlands identified, *inter alia*, collaboration and communication problems among health care providers and employers as barriers for the application of a guideline for occupational health physicians on how to deal with mental health problems [22]. Subsequently, special training was provided for the occupational health physicians to enhance guideline adherence and hence improve return to work for the employees they treated. The randomized controlled trial did not result in the earlier return to work of employees who were guided by the trained occupational health physicians [23]. However, taking workplace conditions into consideration during psychotherapy treatment is considered important for therapy planning [24] and has been shown to improve patient’s work ability [25,26]. 

Occupational health physicians may detect the early signs of mental disorders in employees and refer them for further care [27,28,29,30]. They are familiar with the work environment of employees and, furthermore, experts in the prevention of work-related diseases. This information is important for both primary care physicians, who are often the first medical contact for people with mental disorders [31], and psychotherapists, who are involved in planning and performing the treatment. In addition, occupational health physicians need to have information about the work abilities and operational capabilities of sick employees in order to provide support for them within the company. Collaborative care, offering psychotherapeutic consultation in the workplace for individuals who are not yet sick-listed, has been shown to address patients earlier in the course of disease [32], while being as effective as established care [33]. Moreover, from a health economic perspective, the screening and treatment of depression in the workplace or workplace strategies targeting suicidal behavior or self-harm appear to be very promising [34,35].

Despite the compelling need for collaboration in mental health care, implementation remains a challenge [16,22] and training only one of the groups of collaborators appears to be insufficient [23]. Concentrating on primary care physicians and attuning them to anxiety may even be detrimental, as it reduced the labor force participation rates reported in a study from Yelin et al. in 1996 [36]. Moreover, clear concepts, such as comprehensive guidelines, are lacking [19] in the German health care system. This is why we investigated the collaboration between occupational health physicians, primary care physicians and outpatient psychotherapists in cases of common mental disorders in this study. There are more stakeholders to collaborate with at this interface, for example social workers within a company. Moreover, rehabilitational psychosomatic medicine regulated by the Social Security Statute Book IX in Germany is an important stakeholder in this field. Data on individuals who should be particularly engaged in the prevention of common mental disorders are under preparation for submission [37], as well as data on perspectives of human resource representatives and employees [38]. However, to start with a well-defined group, we focused on occupational health care and statutory medical and psychological health care providers in the outpatient sector and thus only medical care regulated by the Social Security Statute Book V (acute care). The main questions to be answered are: Does any kind of knowledge exchange exist between the different professional groups regarding the prevention of common mental disorders in employees? Which barriers exist? How is collaboration evaluated in this field? And is there a need and interest in further training on the topic?

## 2. Materials and Methods

In July 2014, the six-page questionnaire was sent out in Baden-Württemberg (Germany). The Association of Statutory Health Insurance Physicians organizes all outpatient health care on contract to the Social Security Statute Book V in Germany. Organized in each federal state of Germany, it represents in total over 20,000 physicians and psychotherapists of all fields in Baden-Württemberg. Via their register, the questionnaire was sent to 1000 primary care physicians and 700 resident psychotherapists (simple random selection from Internet address database www.arztsuche-bw.de). Primary care physicians include specialists in general practice, specialists in internal medicine and practical physicians without specialization working in general practice. Psychotherapists can be medical or psychological psychotherapists. Medical psychotherapists can be specialists in psychiatry or in psychosomatic medicine. Specialists from other medical disciplines can also achieve registration as a psychotherapist via further educational training (‘specialized psychotherapy’ accredited by the Medical Chamber). Another further educational training ‘psychosomatic basic care’ does not lead to accreditation as a psychotherapist and thus did not qualify for inclusion in the group of psychotherapists in our sample. There was no systematic evaluation of whether primary care physicians and occupational health physicians finished ‘psychosomatic basic care’.

Furthermore, the questionnaire was sent to 450 occupational health physicians (all members of the Association of German Company and Factory Doctors (VDBW) in the federal state of Baden-Württemberg). In Germany, occupational health physicians have a focus on prevention. Mostly, they are employed by a company and provide screening procedures, vaccinations etc., basic medical counselling and support in reintegration after sickness absence to the employees of the contracted company. They arbitrate between employees and employers with regard to working conditions. An occupational health physician in Germany can be employed directly by a company or by a medicine service company that offers the service. Self-employment is another option when working in private practice or directly in a company or a medicine service company.

The questionnaire was based on the current literature, the results of interviews and standardized surveys of our previous studies on the interface between occupational health physicians and psychotherapists or primary care physicians, respectively [27,39,40], and on our own research into psychosomatic consultation in the workplace and work-related common mental disorders [28,29,30]. Further information on the concept and development is available in Michaelis et al. [37]. 

The research questions were operationalized as follows:

- Experience of direct contact with the respective other professional groups (1 item, yes - no) and number of patients/employees.
- Reasons for non-conclusion in the case of active contact attempts (three items: contact partner could not be reached, contact/contact attempts were too time-consuming, patient/employee did not agree to contact).
- Evaluation of cooperation—two items: last cooperation and all experienced cooperation (rating from 1 = very good to 6 = very bad) and evaluating the importance of collaboration with the other two professions (four specified response options from very important to very unimportant).
- Evaluation of interest in further educational training (four specified response options from very high to very low interest).

### Statistical Methods

The evaluation was made descriptively and by using (a) Chi^2^ and (b) Mann–Whitney-U (MW-U) tests to analyze group differences. Respective effect sizes were consistency coefficient phi and contingency coefficient CC (Chi^2^ test) and ‘w’ (MW-U test, formula: U/root_(number of valid answers)_). Effect sizes for both tests were defined as ≥0.1 as small, ≥0.3 as moderate, and ≥0.5 as high [41]. Means will be communicated including standard deviations (SD). A non-responder analysis was carried out with the following predictors available in the respective databases:

- Occupational health physicians: age, sex.
- Primary care physicians: sex, size of the city (indicated by postal code).
- Psychotherapists: sex, size of the city, professional differences (psychological psychotherapist vs. physician working as a psychotherapist).

More details regarding the design of the study and the collectives are available in Michaelis et al. [37].

## 3. Results

### 3.1. Sample Description

The response rate of the questionnaire was 30% (*n* = 133) among occupational health physicians, 14% (*n* = 136) among primary care physicians and 27% (*n* = 186) among psychotherapists. The quality of the data was good (generally less than 5% of information missing). 

To examine the impact of the non-response bias on our results, a non-responder analysis was conducted. It revealed more participating women in occupational health physicians but not for the other professions. In the primary care physician (but not psychotherapist) group, the response rate was higher among respondents practicing in smaller than in larger cities, but with low effect size (*p*_(chi2) =_ 0.009, phi = 0.08). No age differences between responders and non-responders were found among occupational health physicians and no professional differences among psychotherapists.

More than 50% (*n* = 70 + 81 = 151 out of 269 valid answers) of participating occupational health physicians and primary care physicians were male, whereas only 30% (*n* = 56) of psychotherapists were male, in line with the demographics of the surveyed sample. The mean age of all participants was 53 years (SD 9). Professional experience ranged between 14 and 26 years between the surveyed professions. The responding occupational health physicians and psychotherapists practiced predominantly (detailed information in Table 1) in urban centers, whereas this was only true for 30% (*n* = 42) of the responding primary care physicians. Most occupational health care physicians (*n* = 108 out of 115, 81%) attended large enterprises with 250 and more employees.

Experiences with mental health issues during the training for the specialization in their field were reported by 34 (26%) occupational health physicians and 45 (33%) primary care physicians.

Addressing the topic ‘prevention of mental disorders in the workplace’ in theory was reported by 65 (48%) primary care physicians and 104 (56%) psychotherapists.

Experiences with a formalized return to work process named stepwise reintegration of employees (German Social Code §28 SGB IX/§74 SGB V) or with occupational reintegration management according to §84 SGB IX was reported by 126 (96%) occupational health physicians, 99 (80%) primary care physicians and *n* = 104 (56%) psychotherapists.

### 3.2. Extent of Collaborations between Three Professions to Prevent Common Mental Disorders in Employees

**Figure 1:**
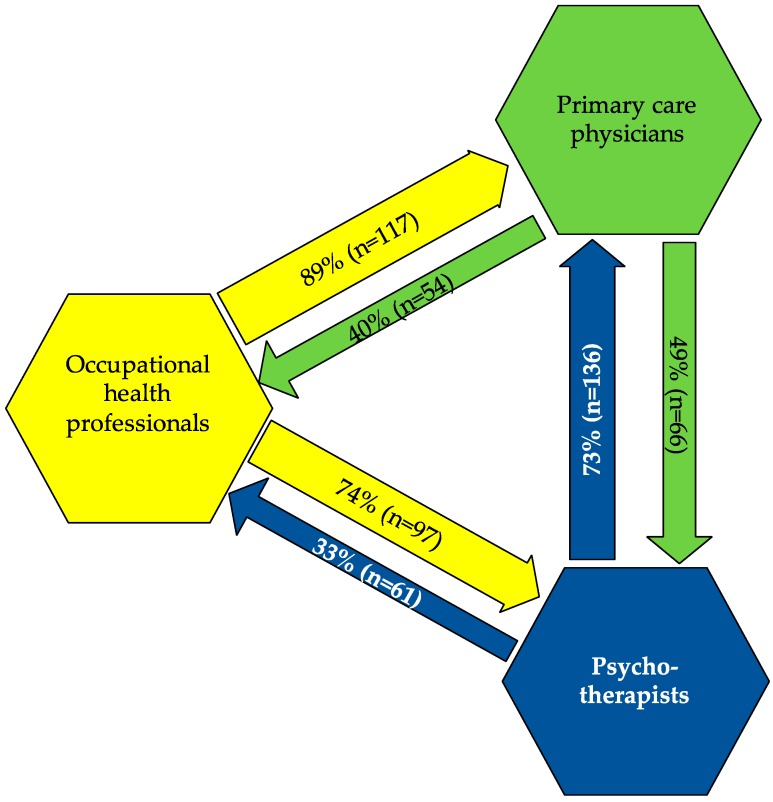
Experience of a successful approach to get in contact with the respective other professions regarding primary, secondary or tertiary prevention of common mental disorders in employees from three perspectives. Percentages and numbers are based on the valid answers.

The experience of a successful approach to make contact regarding the primary, secondary or tertiary prevention of common mental disorders in employees between occupational health physicians, primary care physicians and psychotherapeutic colleagues is displayed in Figure 1.

The occupational health physicians are the professional group including the highest number of individuals in contact with other professional groups due to the referral of a patient with a mental disorder. Nearly all of them (*n* = 117, 89%) reported contact with the primary care physician and 73% (*n* = 97) of them with psychotherapists. However, only 40% (*n* = 54) of primary care physicians and 33% (*n* = 61) of psychotherapists stated that they have contacted an occupational health physician regarding the mental disorder of a patient.

Among psychotherapists and, even more significantly, among primary care physicians, there was a positive correlation with the indicated intensity of their interest in the topic of ‘prevention of mental disorders at the workplace’ and previous contact (*p* = 0.041 and 0.001, CC = 0.21 and 0.33; no figure).

Half of the surveyed individuals made contact with a member of the respective other group in relation to one to four patients with a mental disorder within the last two years (median). Depending on the group, the median was between one (psychotherapists making contact with an occupational health physician) and four (occupational health physicians making contact with primary care physicians) cases. The average number of patients with a mental disorder leading to contact with the respective other professional groups within the last 24 months is detailed in Table 2.

### 3.3. Barriers of Collaboration between Three Professions to Prevent Common Mental Disorders in Employees

Generally, the surveyed individuals with appropriate experience reported self-initiated contacts. Questioned about possible reasons for unsuccessful attempts at establishing contact, 15% (*n* = 10) of primary care physicians and 33% (*n* = 31) of occupational health physicians with a negative experience mentioned the lack of accessibility to psychotherapists (Table 3). Occupational health physicians regarded the attempts at contacting primary care physicians (*n* = 15, 13%) and psychotherapists (*n* = 13, 14%) as too time-consuming (Table 3). For the occupational health physicians, the lack of consent to contact the other profession by patients was the reason to not contact a primary care physician for 12% (*n* = 14) and not to contact the psychotherapist for 17% (*n* = 16) (Table 3). This kind of barrier to contact was only reported by 2% (*n* = 1) of the responding primary care physicians and 3% (*n* = 2) of the responding psychotherapists.

### 3.4. Evaluation of Collaborations between Three Professions to Prevent Common Mental Disorders in Employees

All of the surveyed individuals rated their last contact within the past two years with an average of 2–3 out of 6 (with 1 being the best). There were no differences within the respective collaborations between the professions (occupational health physicians and primary care physicians *p* = 0.07, occupational health physicians and psychotherapists *p* = 0.24, primary care physicians and psychotherapists *p* = 0.15, Mann-Whitney U-test).

Of the surveyed 455 individuals in three professional groups, 87–94% indicated that improving bilateral collaboration was ‘very important’ or ‘rather important’ for the prevention and treatment of mental disorders in employees.

**Figure 2:**
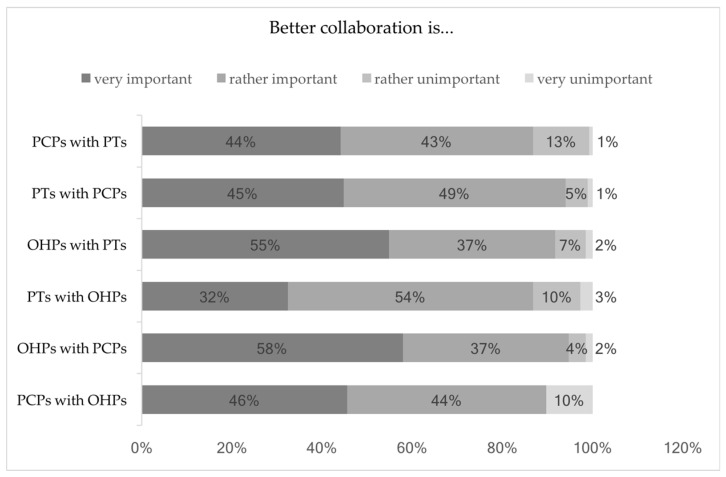
Importance of a better collaboration for the prevention of common mental disorders. Abbreviations: OHPs = occupational health physicians, *n* = 133, PCPs = primary care physicians, *n* = 136, PTs = psychotherapists, *n* = 186.

Less than half of the primary care physicians found a better collaboration with psychotherapists to be ‘very important’ and also vice versa (44% and 45%, *n* = 59 and 84). The same was true for 31 primary care physicians addressing occupational health physicians (46%), whereas the latter group rated the collaboration with primary care physicians and psychotherapists significantly more important (*n* = 77 and 73, 58% and 55%, *p* = 0.030 and 0.000, *w* = 0.13 and 0.22). Details are presented in Figure 2.

### 3.5. Significance of Further Educational Training at the Interface of Three Professions to Prevent Common Mental Disorders in Employees

**Figure 3:**
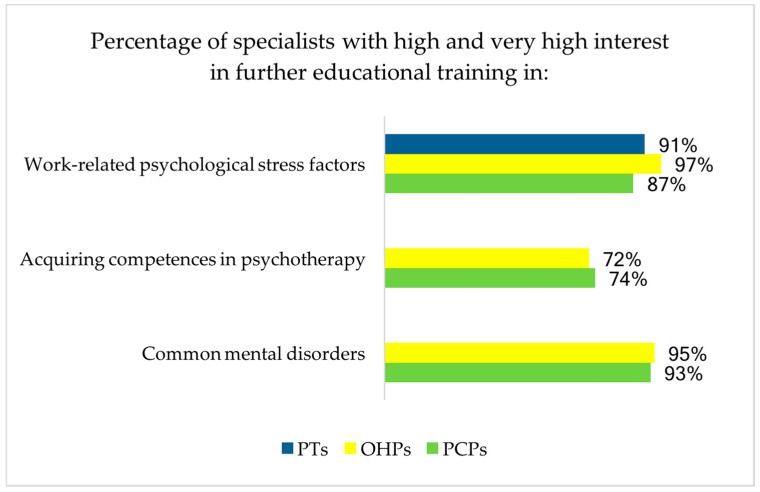
Need for further educational training (FET) for each profession. Range of answers from 1 (very unimportant) to 4 (very important). Abbreviations: OHPs = occupational health physicians (*n* = 131–132), PCPs = primary care physicians (*n* = 134–135), PTs = psychotherapists (*n* = 184). FET in “acquiring competences in psychotherapy” and “common mental disorders” is not applicable to psychotherapists as this is a main part of their common educational training.

Psychotherapists, primary care physicians and occupational health physicians homogenously reported motivation to obtain more knowledge about work-related stress factors (see Figure 3). More than 70% of occupational health physicians and primary care physicians stated their interest in acquiring more psychotherapeutic competencies and reported a very strong interest in more specific knowledge about common mental disorders (displayed in Figure 3).

## 4. Discussion

For the first time, our study explored the actual extent and evaluation of the collaboration of 455 professionals from different disciplines and sectors dealing with the prevention of mental disorders in employees. Despite the fact that almost all of the surveyed individuals attributed great importance to a better collaboration at the interfaces of care, our data show that many primary care physicians and psychotherapists never had contact with an occupational health physician.

The significance of further educational training, which would enhance each individual’s knowledge on the topic of mental disorders and work, was emphasized by all professional groups. A joint interdisciplinary approach and implementation of such events would be a promising strategy.

### 4.1. Cooperation between Occupational Health Physicians and Primary Care Physicians

Knowledge about the workplace is useful for primary care physicians when trying to help employees with mental disorders to maintain their ability to work. Contact with the occupational health physician in cases such as this can be essential [27,39]. According to the results of our study, these contacts are often part of the occupational health physicians’ ‘core business’, while the primary care physicians’ reports of fewer experiences of contact support the results of other studies. In these other studies, explanations for the distance between professions included a lack of primary care physicians’ knowledge of the work performed by occupational health physicians, reservations about the responsibilities that occupational health physicians have and doubts about how independent of the employers’ interests they are [39,40,42].

### 4.2. Cooperation between Occupational Health Physicians and Psychotherapists

Resident psychotherapists and psychotherapeutically-oriented physicians in our survey sample do not show much of a presence or accessibility at the interface to the working world, in line with a recent qualitative investigation which found that role models of physicians in mental health care did not ‘(…) seem to include cooperation with other disciplines as a core task’ [16].

Little is known about whether a general fear of misuse of the information of the patient’s illness or psychotherapeutic treatment obstructs psychotherapists from communicating with other health professionals, e.g., in cases of vocational reintegration. A fear of the misuse of information dealing with mental health problems by other disciplines has been reported by occupational health physicians [22,27] as well as a lack of knowledge about the tasks, concepts and professional framework of the cooperating partners [16,18,43,44].

One important reason for the low level of collaboration of resident physicians and psychotherapists with occupational health physicians—in view of time restraints in providing care to patients—might be the lack of compensation [16,27,40,45]. Motivation and interest in the topic seem to exist according to the results of our study, since almost anyone considers a better collaboration with the occupational health physician to be necessary for the prevention of mental disorders among employees.

The significance of further educational training regarding work-related psychological stress factors and their prevention, as recognized by the professional groups themselves, opens doors for intensified activities in this field. Interfaces for cooperation with occupational health physicians in Germany are already mentioned as part of the Social Code (Social Security Statute Book V and IX) with occupational integration management or stepwise reintegration. The interface is addressed in German AMWF guidelines [46]. Although there is still a lack of systematic collaboration in practice, compared to other countries [20], practical initiatives—in which the collaboration between psychotherapists and occupational health physicians plays an important role, e.g., easily accessible, integrated and fast care concepts such as ‘psychosomatic consultation in the workplace’—are developing and are currently at the beginning of an empirical evaluation [28,29,30,32,33,47].

### 4.3. Cooperation between Primary Care Physicians and Psychotherapists

For years, greater collaboration has been advocated in the fundamental political programs of the professional associations of primary care physicians and psychotherapists (and positively further developed in exemplary model projects) [48]. In our study—in the areas where collaborations take place—these were also rated by all of the surveyed individuals at least as ‘fair’.

However, primary care physicians do not always seem sufficiently trained to recognize the need for the treatment of mental disorders. In addition, they must be aware of the available and appropriate care options [15]. The low degree of professional exchange with psychotherapists, which our data illustrate, may explain the lack of knowledge about relevant care options, even though this interface has now been addressed in selective contracts for several years.

In Germany, a law passed in June 2015 (GKV-VSG 2015) to intensify health care is expected to encourage further steps in this direction. This law mainly focuses on improved communication and more intensive collaboration between physicians and psychotherapists [49]. In the same year, several psychotherapeutic and medical professional associations suggested, for the first time, creating a joint contractually regulated medical care mandate.

There are some limitations of our study. The low survey response rate between 14% and 27% limits the generalizability of our findings, even though other studies in this field also report response rates between 7% and 15% [50,51]. The low response rate, especially from the random sample of primary care physicians (14%), leads us to presume a strong positive selection. Therefore, critical views among primary care physicians in this study might be underrepresented.

Hence, the risk of overestimating results in a positive selected sample is likely. Thus, it can cautiously be assumed that there might be fewer contact experiences in the entire population of professionals. Furthermore, generalizability to other countries and even within Germany is limited, as the study was performed in one of 16 federal states in Germany and should therefore be regarded as only the first step. Furthermore, regarding the participating occupational health physicians, most of them were working at large companies, which indicates a higher affinity to workplace health management. For the primary care physicians and psychotherapists, we assume that very motivated colleagues who are interested in workplace mental health prevention predominantly participated in the study. The assessment of a sample bias was limited by the small number of available variables to control non-responder behavior. Furthermore, there were no common variables in any groups (which in this survey was only sex) as confounders. Another limitation of the quantitative approach and the selection of items that were investigated is that the study does not provide insight into why the study participants got into contact and what kind of information was shared or how the collaborating parties perceived each other. This could be explored in a qualitative approach.

## 5. Conclusions

Collaborating with occupational health physicians appears to be important for primary care physicians and psychotherapists in order to obtain sufficient knowledge regarding possible work-related stress factors, be aware of the resources available to employees with mental disorders and to provide adequate care using evidence-based guidelines. In order to provide optimal care and adjustments to the work environment, occupational health physicians also depend on the patient’s treating physicians and psychotherapists for early intervention and information.

To establish quality-assured cooperation structures in practice, participants need applicable frameworks on an organizational and legal level.

## Figures and Tables

**Table 1:** Sample characteristics.

	OHP ^1^ (*n* = 133)	PCP ^2^ (*n* = 136)	PT ^3^ (*n* = 186)	Differences between Groups, Stat. Test
Age (years, SD), *n* valid	54.9 (8.0), 133	53.7 (8.6), 130	53.9 (8.6), 183	n.s., MW-U-test
Gender (male, *n*/*n* valid)	52.6% (70/133)	59.6% (81/136)	30.1% (56/186)	OHP-PCP n.s., OHP-PT *p* = 0.000, phi = 0.23, PCP-PT *p* = 0.000, phi = 0.29, Chi^2^ test
Professional experience (years working within that specialization or in own practice, SD), *n* valid	26.7 (8.5), 114	18.3 (9.3), 127	13.6 (8.2),182	n.s., MW-U-test
Practicing in urban centers	57.0% (73/128)	30.9% (42/136)	60.0% (111/185)	OHP-PCP *p* = 0.000, phi = 0.26, PCP-PT *p* = 0.000, phi = 0.29; OHP-PT n.s., Chi-^2^ test

^1^ Occupational health physicians (OHPs) including medical specialists and physicians with additional qualification in “Occupational medicine”; ^2^ Primary care Physicians (PCPs) including specialists in general practice, specialists in internal medicine and practical physicians without specialization working in general practice; ^3^ medical (*n* = 65) and psychological psychotherapists (PTs) (*n* = 121) including medical specialists in psychotherapy, psychiatry and psychosomatic medicine.

**Table 2:** Patients with a mental disorder, leading to contact with the respective other professional group within the last 24 months.

Responding Group	Addressed Collaboration Group		
	OHPs	PCPs	PTs
OHP	-	4 13.4 (45.5) *n* = 117/118	2 10.4 (43.7) *n* = 97/97
PCP	2 2.3 (3.0) *n* = 54/54	-	2 4.6 (7.2) *n* = 65/66
PT	1 2.4 (5.2) *n* = 61/61	3 9.5 (41.1) *n* = 61/61	-

Numbers are reported as: median, mean (standard deviation), number of valid cases/number of respondents having reported contact to the respective other group.

**Table 3:** Reasons for actively initiated but unsuccessful contact with members of the other professional groups.

Contact Attempts between…		Reasons for Unsuccessful Contact		
	*n*	Contact Partner Was Not Available % (*n*)	Contact Attempt Was Too Time-Consuming % (*n*)	Patient/Employee Did Not Agree to Contact % (*n*)
occupational health physicians and primary care physicians	115	14% (16)	13% (15)	12% (14)
occupational health physicians and psychotherapists	95	33% (31)	14% (13)	17% (16)
primary care physicians and occupational health physicians	54	9% (5)	7% (4)	2% (1)
primary care physicians and psychotherapists	67	15% (10)	9% (6)	0% (0)
psychotherapists and occupational health physicians	60	3% (2)	2% (1)	3% (2)
psychotherapists and primary care physicians	135	2% (3)	2% (3)	0% (0)

First mentioned is the group seeking contact, second the group being addressed. Multiple answers with three standardized specified reasons (random subset: respondents with negative contact experience).
